# Evaluation of the drug-drug interactions management system for appropriate use of nirmatrelvir/ritonavir: a retrospective observational study

**DOI:** 10.1186/s40780-024-00376-4

**Published:** 2024-09-03

**Authors:** Takeshi Tomida, Takeshi Kimura, Kazuhiro Yamamoto, Atsushi Uda, Yuki Matsumoto, Naoki Tamura, Masashi Iida, Akiko Tanifuji, Kumiko Matsumoto, Naomi Mizuta, Kei Ebisawa, Goh Ohji, Tomohiro Omura, Kentaro Iwata, Ikuko Yano

**Affiliations:** 1 Department of Pharmacy, Kobe University Hospital, 7-5-2 Kusunoki-Cho, Chuo-Ku, Kobe, 650-0017 Japan; 2 Department of Integrated Clinical and Basic Pharmaceutical Sciences, Faculty of Medicine, Dentistry and Pharmaceutical Sciences, Okayama University, Okayama, 700-8558 Japan; 3 Division of Infectious Diseases, Kobe University Hospital, Kobe, 650-0017 Japan

**Keywords:** COVID-19, Nirmatrelvir/ritonavir, Drug–drug interactions, Molnupiravir, Antivirals

## Abstract

**Purpose:**

While nirmatrelvir/ritonavir (NMV-r) has been positioned as a first-line treatment for mild to moderate COVID-19, it has multiple and significant drug-drug interactions (DDIs). The use of NMV-r in Japan has been limited compared to the United States. This study aimed to describe the distribution of DDIs with NMV-r and their management in patients with COVID-19 under the control of a management system for the appropriate use of NMV-r.

**Methods:**

A retrospective observational study was conducted at a Japanese university hospital. The management system included a flowchart for selecting antivirals and a list for reviewing DDI management, based on the National Institutes of Health guidelines and the guidance of the Japanese Society of Pharmaceutical Health Care and Sciences. Patients with mild to moderate COVID-19 and prescribed NMV-r or molnupiravir (MOV) were included. The primary outcome was DDI management practices, including the selected COVID-19 medications. The secondary outcome included the distribution of DDI classification and the 30-day all-cause mortality.

**Results:**

This study included 241 patients (median age of 60 years, 112 [46.5%] females), of whom 126 and 115 received NMV-r and MOV, respectively. Of the 241 patients, 145 (60.2%) received concomitant medications that have DDIs with NMV-r. All 30 patients with severe renal impairment or insufficient details on concomitant medications received MOV. Forty-nine patients with concomitant medications required alternative COVID-19 therapy consideration due to DDIs, of whom 42 (85.7%) patients received MOV. Eighty-one patients had concomitant medications requiring temporary adjustment, of whom 44 (54.3%) patients received NMV-r, and 42 of these patients temporarily adjusted these concomitant medications. Five patients with concomitant medications that can continued by monitoring the effects/adverse effects, of whom 4 (80.0%) patients received NMV-r. Seventy-six patients without concomitant medications requiring DDI management, of whom 71 (93.4%) patients received NMV-r. The 30-day all-cause mortality for eligible patients was 0.9% [95% confidence interval, 0.1–3.1].

**Conclusions:**

Most patients received appropriate antivirals according to the classification of DDIs, and most patients with concomitant medications requiring temporary adjustment received the recommended DDI management. Our management system is effective in promoting the use of NMV-r in the appropriate patients and managing problematic DDIs.

**Supplementary Information:**

The online version contains supplementary material available at 10.1186/s40780-024-00376-4.

## Introduction

The coronavirus disease 2019 (COVID-19) pandemic, caused by severe acute respiratory syndrome coronavirus 2 (SARS-CoV-2), has been a global threat, with over 770 million cumulative cases and 6.95 million cumulative deaths reported worldwide [1]. Antiviral drugs are crucial for COVID-19 treatment. Among them, nirmatrelvir/ritonavir (NMV-r) has demonstrated high efficacy in the clinical trial [2] and is considered the first-line treatment for mild to moderate COVID-19 by the National Institutes of Health (NIH) guidelines [3]. Studies analyzed real-world data since the spread of the SARS-CoV-2 Omicron variant have revealed that NMV-r reduced the risk of hospitalization and death in all patient populations, including those with adequate vaccination status [4, 5]. These remarkable results are different from those on molnupiravir (MOV), another oral antiviral for COVID-19, which demonstrated no effectiveness in the patient population with adequate vaccination status [6, 7].

NMV-r has multiple and significant drug-drug interactions (DDIs), since included ritonavir is a strong inhibitor of cytochrome P450 enzymes (CYPs) and P-glycoprotein (P-gp) [8]. A cohort study estimated that 3,378 (5.4%) of 62,525 eligible patients hospitalized with COVID-19 had contraindications related to DDIs with NMV-r [9]. In a retrospective study involving patients prescribed NMV-r, contraindications due to DDIs were observed in 14.6% of the subjects [10], and clinically significant DDIs were observed in 60–70% of the target patients [11, 12]. Moreover, severe adverse events caused by DDIs with NMV-r have been reported for multiple medications such as tacrolimus, clozapine, and others [13–18]. The complexity of DDI management may hinder NMV-r use. Data through September 2022 in Japan demonstrated that NMV-r was administered in only approximately 44,000 patients, while MOV was administered in approximately 620,000 patients [19]. This differs from the distribution of drug usage in the United States, where NMV-r was given to approximately seven times as many patients as MOV [20]. The previous Japanese COVID-19 treatment guides did not distinguish the strength of recommendations for each antiviral drug [21, 22]. The use of NMV-r, which is more complex to manage DDIs, may have been discouraged in situations where the strength of recommendations for NMV-r or MOV was comparable. Therefore, evaluating the distribution of DDI classification and its actual management and developing a system to safely manage DDIs are necessary for the appropriate use of NMV-r and the prevention of adverse events due to DDIs. However, to the best of our knowledge, no studies have evaluated the effectiveness of the management system for DDIs of NMV-r.

Information on DDIs of NMV-r includes the Emergency Use Authorization fact sheet [23] and the NIH guidelines on DDIs of NMV-r [8]. In Japan, “Guidance on the management of drug–drug interactions with Paxlovid (nirmatrelvir/ritonavir)” was issued by the Japanese Society of Pharmaceutical Health Care and Sciences (JSPHCS) [24]. This guidance was developed based on the fact sheet information [23], the NIH DDI guidelines [8], and other studies on DDIs [25, 26]. Since different drugs are used in different countries, utilizing local resources in addition to global guidelines to review DDIs with NMV-r may minimize the occurrence of inappropriate DDI management.

We have developed the management system for the appropriate use of NMV-r, including DDI management, at Kobe University Hospital. The management system included a flowchart for selecting antivirals in patients with mild to moderate COVID-19 and a list that shows drugs requiring caution for DDIs with NMV-r and recommended DDI management, based on the information resources for DDIs [8, 24]. This observational study aimed to describe the distribution of DDIs with NMV-r and their management in patients with mild to moderate COVID-19 under the control of the management system for the appropriate use of NMV-r in DDI management.

## Methods

### Study design and settings

This retrospective observational study was conducted at Kobe University Hospital, Japan. The study protocol was approved by the Ethical Committee of Kobe University Hospital (No. B230101), and the study was performed in accordance with the Declaration of Helsinki and its amendments. Informed consent was not obtained from individual patients, nevertheless, an opt-out opportunity was set on the hospital’s website. This report followed the Strengthening the Reporting of Observational studies in Epidemiology statement [27].

### The management system for appropriate use of NMV-r

**Fig. 1:**
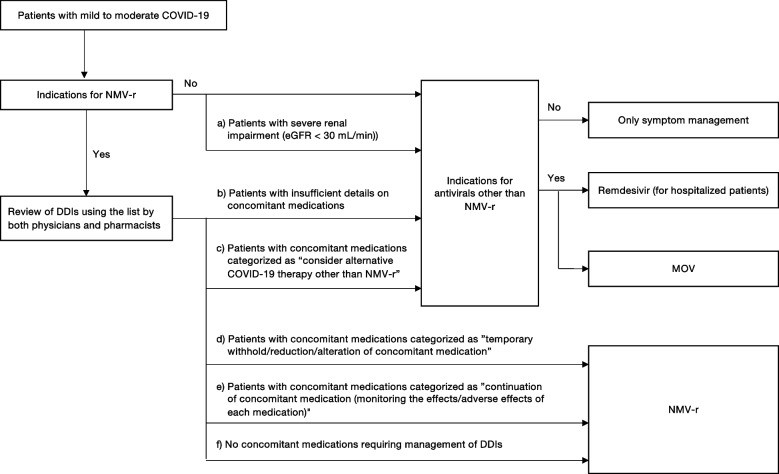
Flowchart for the antiviral selection in patients with mild to moderate COVID-19. COVID-19, the coronavirus disease 2019; DDIs, drug-drug interactions; eGFR, estimated glomerular filtration rate; MOV, molnupiravir; NMV-r, nirmatrelvir/ritonavir

The management system for the appropriate use of NMV-r has been established since March 2022. In establishing the system, we have developed a flowchart for selecting antivirals in patients with mild to moderate COVID-19 (Fig. 1), as well as a list presenting drugs that require caution for DDIs with NMV-r and recommended DDI management. The system consisted of selecting appropriate antivirals and reviewing DDIs with NMV-r using the flowchart and the list. The constructed flowchart and list were disseminated to medical staff through hospital meetings and the website.

The flowchart for selecting antivirals in patients with mild to moderate COVID-19 has been developed under the NIH COVID-19 treatment guidelines [3]. The flowchart shows NMV-r as the first-line antiviral for patients with mild to moderate COVID-19. We recommended considering other antivirals if patients were not indicated for NMV-r, including those with severe renal impairment (estimated glomerular filtration rate [eGFR] of < 30 mL/min) (Fig. 1 (a)). Alternative antivirals other than NMV-r included remdesivir for hospitalized patients or MOV. We only recommended symptom treatments for patients with no indications for other antivirals.

For patients indicated for NMV-r, both physicians and pharmacists reviewed and managed DDIs between NMV-r and other medications taken regularly referring to the list. The list for reviewing DDIs was based on the NIH DDI guidelines [8] and the JSPHCS guidance for DDI management [24]. The JSPHCS DDI guidance lists the drugs that have problematic DDIs with NMV-r, including CYP3A substrates, P-gp substrates, and moderate to high CYP3A inducers, and provides recommended DDI management [24]. Our list specifically described the effects of DDIs in each drug, such as increased blood concentrations of concomitant medications or decreased blood concentrations of NMV and recommended DDI management. We recommended that antivirals other than NMV-r should be considered for patients with insufficient details on concomitant medications (Fig. 1 (b)) or those with concomitant medication categorized as “consider alternative COVID-19 therapy other than NMV-r” (Fig. 1 (c)). The use of NMV-r was recommended in patients for whom adjustment of concomitant medication with DDIs was feasible, such as patients with concomitant medication categorized as “temporary withhold/reduction/alteration of concomitant medication” (Fig. 1 (d)) or “continuation of concomitant medication (monitoring the effects/adverse effects of each medication)” (Fig. 1 (e)), and patients without concomitant medications requiring DDI management (Fig. 1 (f)). Pharmacists inquired of physicians in the presence of problems in DDI management. An Excel file was developed to identify concomitant medications with DDIs and their recommended management and was used by the pharmacists to check for DDIs.

### Study subjects and sample size

Outpatients and inpatients with mild to moderate COVID-19 aged ≥ 18 years who were at risk for severe COVID-19 and who were prescribed NMV-r or MOV from April 1, 2022 to March 31, 2023, were included. Patients who used remdesivir during the study period were not included, because they had severe COVID-19 as well as mild to moderate diseases. A cohort study estimated that 5.4% out of 62,525 eligible inpatients with COVID-19 had contraindications due to DDIs with NMV-r [9]. The present study aimed to evaluate the management for all problematic DDIs with NMV-r, including contraindications to the concomitant use. Patients receiving MOV were also included because MOV may have been selected in patients with problematic DDIs with NMV-r. If we estimate that 10% of eligible adult patients with mild to moderate COVID-19 have problematic DDIs with NMV-r, 225 patients were needed to detect at a two-sided significance level of 5% and power of 80%. Therefore, the sample size was set at 240 because the analysis was expected to exclude a small number of patients.

### Outcomes

The primary outcome was to describe the actual DDI management practices. Eligible patients were categorized following their baseline characteristics or recommended DDI management in their concomitant medications, and the actual DDI management practices, including the selected COVID-19 medications were evaluated. The secondary outcome was the distribution of DDIs with NMV-r, which evaluated the classification of DDIs for concomitant medications and the number of DDIs per patient. Other secondary outcomes included the proportion of patients who require oxygen therapy within 30 days related to COVID-19, the proportion of patients requiring ventilatory support within 30 days, and the 30-day all-cause mortality. These outcomes were calculated for patients in whom each event occurred within 30 days of antiviral initiation. Baseline characteristics and outcomes were retrospectively assessed via electronic medical records. Renal functions of eligible patients were assessed using eGFR (mL/min/1.73 m^2^), as the height or weight of some patients were retrospectively unavailable from their electronic medical records. Baseline risk factors for severe COVID-19 were evaluated as listed in the COVID-19 clinical practice guide in Japan [21]. The concomitant medications of the included patients were evaluated for the regularly taken medications that were reviewed by the pharmacist or physician during the treatment period of antivirals for COVID-19. The concomitant medications that fall under DDIs with NMV-r were detected based on the JSPHCS DDI guidance [24]. As-needed medications, topical medications, or inhalants were excluded from the evaluation. The recommended DDI management was categorized as “consider alternative COVID-19 therapy other than NMV-r,” “temporary withhold/reduction/alteration of concomitant medication,” or “continuation of concomitant medication (monitoring the effects/adverse effects of each medication),” according to our list.

## Results

### Patient characteristics

Table 1 shows the baseline characteristics of the included patients. A total of 241 patients were included (median age of 60 years, 112 [46.5%] females, and median risk factors of three). Risk factors that more than 40% of eligible patients had included patients aged over 65 years, malignancy, hypertension, and use of immunosuppressants. Number of hospitalized patients was 44 (18.3%). Two or more doses of vaccination for SARS-CoV-2 had been administered to 170 (70.5%) patients. Of the eligible patients, 126 (52.3%) patients were prescribed NMV-r (NMV-r group) and 115 (47.7%) patients were prescribed MOV (MOV group).

**Table 1:** Baseline characteristics of the study patients

Category^a^	Total (*n* = 241)
Age (years)	60 [45.0–73.0]
Female	112 (46.5%)
eGFR (mL/min/1.73 m^2^)	70.0 [50.9–85.1]
Unknown	9
Number of severity risk factors	3 [2–5]
≥ 65 years of age	104 (43.2%)
Malignancy	98 (40.7%)
Chronic respiratory disease	28 (11.6%)
Chronic kidney disease	80 (33.2%)
Diabetes mellitus	53 (22.0%)
Hypertension	104 (43.2%)
Hyperlipidemia	71 (29.5%)
Cardiovascular disease	53 (22.0%)
Cerebrovascular disease	15 (6.2%)
Body mass index ≥ 30 kg/m^2^	15 (6.2%)
Smoking habit (within the past 30 days and more than 100 cigarettes in a lifetime)	20 (8.3%)
Prior solid-organ transplant	17 (7.1%)
Late pregnancy	3 (1.2%)
Use of immunosuppressants (including cancer chemotherapy)	104 (43.2%)
HIV infection (CD4 < 200 cells/mm^3^)	1 (0.4%)
Hospitalized patients	44 (18.3%)
Vaccination status	
0–1	44 (18.3%)
≥ 2	170 (70.5%)
Unknown	27 (11.2%)
Prescribed COVID-19 medication	
NMV-r	126 (52.3%)
MOV	115 (47.7%)

*COVID-19* the coronavirus disease 2019, *eGFR* estimated glomerular filtration rate, *HIV* human immunodeficiency virus, *MOV* molnupiravir, *NMV-r* nirmatrelvir/ritonavir

^a^Median [min–max] or n (%)

### Management of DDIs

**Fig. 2:**
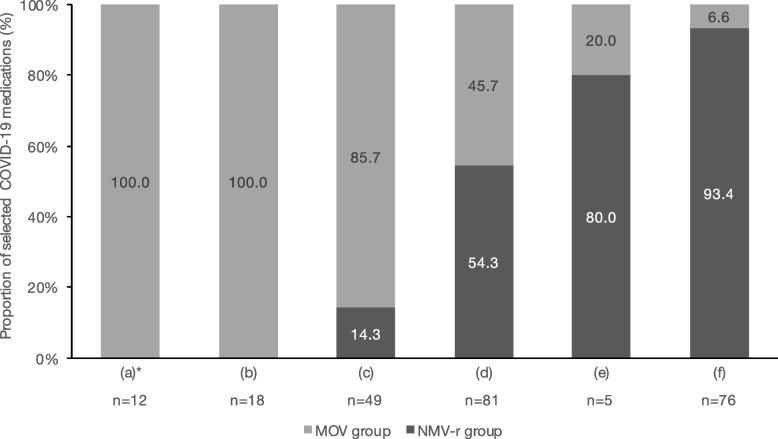
Proportion of selected COVID-19 medications according to patient characteristics or recommended DDI management. **a** Patients with reduced renal function (eGFR of < 30 mL/min/1.73m^2^), (**b**) patients with insufficient details on concomitant medications, (**c**) patients with concomitant medications categorized as “consider alternative COVID-19 therapy other than NMV-r,” (**d**) patients with concomitant medications categorized as “temporary withhold/reduction/alteration of concomitant medication,” (**e**) patients with concomitant medications categorized as “continuation of concomitant medication (monitoring the effects/adverse effects of each medication),” and (**f**) patients without concomitant medications requiring DDI management. COVID-19, the coronavirus disease 2019; DDIs, drug-drug interactions; MOV, molnupiravir; NMV-r, nirmatrelvir/ritonavir. * Classification (**a**) included 10 patients who had concomitant medications that have DDIs with NMV-r, 5 of whom had concomitant medications categorized as “consider alternative COVID-19 therapy other than NMV-r”

Figure 2 shows the proportion of selected COVID-19 medications according to the patient characteristics or recommended DDI management. Of the 241 eligible patients, 12 demonstrated reduced renal function (eGFR < 30 mL/min/1.73m^2^) (a) and 18 had insufficient details on concomitant medications (b), and all of these patients were prescribed MOV. Of the remaining patients, 49 had concomitant medications categorized as “consider alternative COVID-19 therapy other than NMV-r” (c), and 7 (14.3%) of these patients received NMV-r and 42 (85.7%) received MOV. From classification of (c) to (f), the proportion of patients who received NMV-r gradually increased, with 71 (93.4%) patients without concomitant medications requiring DDI management received NMV-r. Patients classified as (a) included 10 patients who had concomitant medications having DDIs with NMV-r, 5 of whom had concomitant medications categorized as “consider alternative COVID-19 therapy other than NMV-r.”

Table 2 demonstrates the actual DDI management practices in the NMV-r group. Of the 7 patients with concomitant medications categorized as “consider alternative COVID-19 therapy other than NMV-r,” 6 (85.7%) were managed with temporary withhold/reduction/alteration of concomitant medications. Of the 44 patients with concomitant medications categorized as “temporary withhold/reduction/alteration of concomitant medication,” 42 (95.5%) were actually adjusted their concomitant medications temporarily.

**Table 2:** Actual management practices of DDIs in the NMV-r group according to the recommended DDI management

Actual DDI management practices according to the recommended DDI management	n (%)
Total	126
(a) Patients with reduced renal function (eGFR < 30 mL/min/1.73m^2^)	0
(b) Patients with insufficient details on concomitant medications	0
(c) Patients with concomitant medications categorized as “consider alternative COVID-19 therapy other than NMV-r”	7
Temporary withhold/reduction/alteration of concomitant medications	6 (85.7%)
Details of management were unknown	1 (14.3%)
(d) Patients with concomitant medications categorized as “temporary withhold/reduction/alteration of concomitant medication”	44
Temporary withhold/reduction/alteration of concomitant medications	42 (95.5)
Continuation of concomitant medication (monitoring the effects/adverse effects of each medication)	2 (4.5)
(e) Patients with concomitant medications categorized as “continuation of concomitant medication (monitoring the effects/adverse effects of each medication)”	4
Temporary withhold/reduction/alteration of concomitant medications	3 (75.0)
Continuation of concomitant medication (monitoring the effects/adverse effects of each medication)	1 (25.0)
(f) Patients without concomitant medications requiring DDI management	71
Temporary withhold/reduction/alteration of concomitant medications	3 (4.2)
No management for DDIs	68 (95.8)

*COVID-19* the coronavirus disease 2019, *DDI* drug-drug interactions, *eGFR* estimated glomerular filtration rate, *NMV-r* nirmatrelvir/ritonavir

### DDIs with NMV-r

**Fig. 3:**
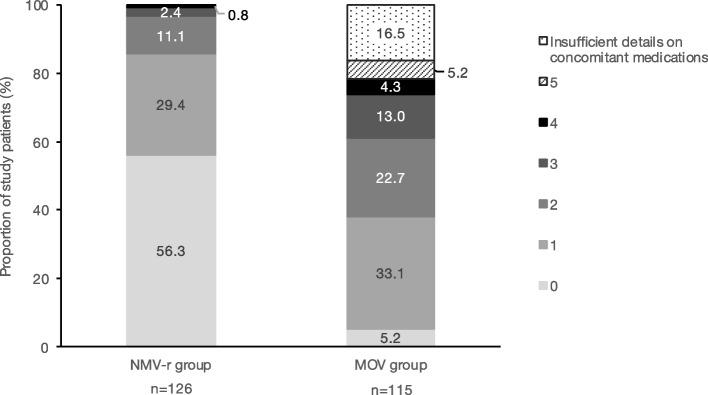
Classification of the included patients by the number of concomitant medications that have DDIs with NMV-r. Each number in the legend indicates the number of concomitant medications that have DDIs with NMV-r. Patients with insufficient details on concomitant medications were separately classified. DDIs, drug-drug interactions; MOV, molnupiravir; NMV-r, nirmatrelvir/ritonavir

Overall, 145 (60.2%) patients had concomitant medications with DDIs with NMV-r (10 patients of (a), (c), (d), and (e) of Fig. 2). Figure 3 shows the classification of included patients by the number of concomitant medications that have DDIs with NMV-r. Of the NMV-r group, 43.7% had one or more concomitant medications that have DDIs with NMV-r. Of the MOV group, 78.3% had one or more and 45.2% had two or more concomitant medications that have DDIs with NMV-r. The classification of concomitant medications that have DDIs with NMV-r under the recommended DDI management is shown in Table 3. Medications categorized as “consider alternative COVID-19 therapy other than NMV-r” included immunosuppressants, antiepileptics, and anticoagulants. Among the medications categorized as “temporary withhold/reduction/alteration of concomitant medication,” calcium channel blockers, 3-hydroxy-3-methylglutaryl-coenzyme A reductase inhibitors, and sedative/hypnotic drugs were frequently prescribed medications. Medications categorized as “continuation of concomitant medication (monitoring the effects/adverse effects of each medication)” included tramadol and fluconazole. Twenty-five (10.4%) patients had concomitant medications contraindicated with NMV-r, including 6 and 19 in the NMV-r and MOV groups, respectively. One patient in the MOV group had two concomitant medications contraindicated with NMV-r.

**Table 3:** Classification of concomitant medications that have DDIs with NMV-r under the recommended DDI management

Classification of concomitant medications			Total	NMV-r group	MOV group
Consider alternative COVID-19 therapy			68	7	61
Immunosuppressants		Tacrolimus	25	1	24
		Cyclosporine	7	0	7
		Everolimus	5	0	5
Antiepileptics		Clonazepam	6	1	5
		Clobazam	2	0	2
		Carbamazepine^a^	1	0	1
Anticoagulants		Warfarin	5	2	3
		Rivaroxaban^a^	4	1	3
Vasopressin antagonists		Tolvaptan	4	0	4
Antineoplastic and immunomodulating agents		Nilotinib	1	1	0
		Tamoxifen	1	0	1
		Toremifene	1	0	1
Antiplatelet agents		Clopidogrel	2	0	2
		Cilostazol	1	1	0
Antiarrhythmics		Amiodarone^a^	1	0	1
		Bepridil^a^	1	0	1
Antimycobacterials		Rifampicin^a^	1	0	1
Temporary withhold/reduction/alteration of concomitant medication			184	62	122
Calcium channel blockers		Amlodipine	48	19	29
		Nifedipine	14	3	11
		Diltiazem	2	1	1
		Verapamil	1	0	1
HMG-CoA reductase inhibitors		Rosuvastatin	19	4	15
		Atorvastatin	17	7	10
Hypnotics and sedatives		Etizolam	8	5	3
		Suvorexant^a^	6	1	5
		Brotizolam	5	3	2
		Lenborexant	3	0	3
		Alprazolam	2	1	1
		Triazolam^a^	1	0	1
Anticoagulants		Edoxaban	7	1	6
		Apixaban	3	0	3
		Dabigatran	1	1	0
Alpha-adrenoreceptor antagonists		Tamsulosin	4	2	2
		Silodosin	3	1	2
Antidepressants		Trazodone	6	0	6
Antipsychotics		Aripiprazole	4	0	4
		Quetiapine	2	0	2
Antigout agents		Colchicine^a^	5	1	4
Antihistamines		Fexofenadine	4	0	4
		Lupatadine	1	1	0
Aldosterone antagonists		Eplerenone^a^	5	2	3
Opioids		Oxycodone	3	2	1
		Fentanyl	1	0	1
Phosphodiesterase-5 Enzyme Inhibitors		Tadalafil	2	1	1
Antibacterials		Clarithromycin	2	2	0
Corticosteroids		Dexamethasone	2	1	1
Antipropulsives		Loperamide	2	2	0
Antimigraine agents		Eletriptan^a^	1	1	0
Continuation of concomitant medication (monitoring the effects/adverse effects of each medication)			13	6	7
Opioids		Tramadol	5	1	4
Antifungals		Fluconazole	4	4	0
Antiepileptics		Valproic acid	2	0	2
Antivirals for the treatment of HIV infections		BIC/TAF/FTC	1	1	0
		Darunavir	1	0	1

*BIC/TAF/FTC* bictegravir/tenofovir alafenamide/emtricitabine, *COVID-19* the coronavirus disease 2019, *DDIs* drug-drug interactions, *HIV* human immunodeficiency virus, *HMG-CoA* 3-hydroxy-3-methylglutaryl-coenzyme A, *NMV-r* nirmatrelvir/ritonavir

^a^Drugs contraindicated with NMV-r

### Clinical outcomes

The proportion of patients requiring oxygen therapy within 30 days related to COVID-19 was 6.3%, 3.4% and 9.5% in the total patients, NMV-r and MOV groups, respectively (Table 4). The proportion of patients requiring ventilatory support within 30 days and the 30-day all-cause mortality ranged under 1% and 2%, respectively, in the total patients and both groups. Baseline characteristics of the patients in the NMV-r and MOV groups were shown in the Table S1.

**Table 4:** Clinical outcomes of the included patients

Clinical outcomes	Total (*n* = 241)		NMV-r group (*n* = 126)		MOV group (*n* = 115)	
	n	% [95%CI]	n	% [95%CI]	n	% [95%CI]
Patients requiring oxygen therapy within 30 days related to COVID-19	14	6.3 [3.5–10.4]	4	3.4 [0.9–8.5]	10	9.5 [4.7–16.8]
Unknown or no subjects^a^	19		9		10	
Patients requiring ventilatory support within 30 days	1	0.4 [0.1–2.4]	0	0.0 [0.0–3.0]	1	1.0 [0.02–5.2]
Unknown^b^	15		5		10	
30-day all-cause mortality	2	0.9 [0.1–3.1]	0	0.0 [0.0–3.0]	2	1.9 [0.2–6.5]
Unknown^b^	10		3		7	

*CI* confidence interval, *COVID-19* the coronavirus disease 2019, *MOV* molnupiravir, *NMV-r* nirmatrelvir/ritonavir

^a^Patients with unknown clinical outcomes or those who received oxygen therapy before initiating antivirals were excluded

^b^Patients with unknown clinical outcomes were excluded

## Discussion

Our study suggested that the management system for the appropriate use of NMV-r facilitated the use of appropriate antivirals following the classification of DDIs and the implementation of recommended DDI management. More than 85% of patients with DDIs requiring the consideration of alternative COVID-19 therapy other than NMV-r received MOV (Fig. 2 (c)). The proportion of patients receiving NMV-r increased according to the classification of DDI management. In the NMV-r group, 95.5% of patients with concomitant medications categorized as “temporary withhold/reduction/alteration of concomitant medication” (Table 2 (d)) adhered the recommended DDI management. Even when the management that differed from the recommendations in our list was selected, safe DDI managements, such as temporary withholding or adjustment of concomitant medications, were implemented. Various adverse events caused by DDIs with NMV-r have been reported [13–18]. For immunosuppressants or cardiovascular drugs, strategies for their adjustment when used in combination with NMV-r have been proposed [28, 29]. However, no studies have evaluated comprehensive management for DDIs of NMV-r. In addition, the use of NMV-r in Japan was only 7% of that of MOV, which differs from the usage in the United States [19, 20]. In the present study, NMV-r was used in more patients than MOV and most patients with problematic DDIs received the recommended DDI management, suggesting that the management system contributed to these results. In our system, both physicians and pharmacists carefully reviewed DDIs using the constructed list, and the DDI management included those implemented following the suggestion of pharmacists. The dissemination of the list that describes the recommended DDI management and the establishment of a multidisciplinary system for reviewing DDIs may have contributed to the appropriate DDI management.

Immunosuppressants such as tacrolimus or cyclosporine were the most common concomitant medications categorized as “consider alternative COVID-19 therapy other than NMV-r”, and all patients except one case received MOV in this study. Several adverse events associated with markedly elevated blood concentrations of tacrolimus in combination with NMV-r have been reported [13–16], and management should be implemented to avoid concomitant administration of these medications. NIH guidelines classify immunosuppressants as “temporarily withhold concomitant medication” and warn that these drugs should not be used if therapeutic drug monitoring (TDM) cannot be closely conducted [8]. Another study recommended the discontinuation of tacrolimus during the treatment period of NMV-r, then resumed with dose adjustment while conducting TDM [30]. Visiting the hospital frequently and conducting TDM closely would be difficult for outpatients with mild COVID-19 due to isolation measures. Thus, immunosuppressants were categorized as “consider alternative COVID-19 therapy other than NMV-r” in our list. Other concomitant medications categorized as “consider alternative COVID-19 therapy other than NMV-r” included clonazepam and warfarin in this study. We differently categorized these drugs from the NIH guidelines [8] because frequently performing TDM or laboratory tests is difficult, similarly as in the case of immunosuppressants. A previous study reported a case series of changes in the international normalized ratio with the combination of warfarin and NMV-r [31]. When drugs that require monitoring of blood drug concentrations or pharmacological indicators are used in combination with NMV-r, a setting that allows close monitoring of the effects of DDIs is required.

Of all eligible patients in this study, 60.2% had concomitant medications with DDIs with NMV-r. In large-scale observational studies involving patients with COVID-19 or those with risk factors for severe COVID-19, the prevalence of DDIs has been simulated to be 5–20% [9, 32, 33]. Meanwhile, 60–70% of eligible patients had clinically significant DDIs in single-center retrospective studies involving patients prescribed NMV-r [11, 12]. Our facility is a university hospital and may have had more patients with risk factors for severe COVID-19, such as malignancy or prior solid-organ transplantation, and patients with concomitant medications that cause problematic DDIs with NMV-r. In addition, our constructed list was based on the JSPHCS DDI guidance [24] as well as the NIH DDI guidelines [8], thus drugs, such as etizolam or brotizolam, which are hypnotics and not listed in the NIH DDI guidelines [8], could be also detected. Although 60.2% of eligible patients had DDIs with NMV-r, only 22% (i.e., 5 patients of (a) and (c) of Fig. 2) of them had concomitant medications that required the consideration of alternative COVID-19 therapy other than NMV-r due to DDIs. Although the use of NMV-r in Japan has been substantially limited compared to the United States [19, 20], potentially more patients in Japan could benefit from the use of NMV-r by managing DDIs appropriately, and there is a need to establish a system to promote the appropriate use of NMV-r, as in this study.

In the MOV group, the sum of patients who had two or more concomitant medications that have DDIs with NMV-r was 45.2% in this study (Fig. 3). Patients for whom NMV-r could be selected according to the DDI management system (i.e., (d), (e), and (f) of Fig. 2), included those who had multiple concomitant medications requiring adjustment due to DDIs. A previous study revealed that DDIs with NMV-r were common in older adults with polypharmacy, and many DDIs involved potentially inappropriate medications (PIMs), which were candidates for deprescribing [34]. The use of NMV-r may have been discouraged in patients with polypharmacy due to the wide variety of DDIs, even when there are no concomitant medications that require consideration of alternative drugs. Implementing deprescribing for PIMs and providing a more specific indication for DDI management, such as showing a reduction dose of concomitant medication or safer alternative drugs, may increase adherence to the DDI management system even in patients who had multiple DDIs with NMV-r, and more patients could benefit from the use of NMV-r.

This study evaluated the clinical outcomes of patients with COVID-19 under the control of the management system for the appropriate use of NMV-r. In previous studies of outpatients who received NMV-r with adequate vaccination status, the proportion of hospitalization was 0.5% [95% confidence interval (CI), 0.4–0.5] and the mortality was 0.01% [95%CI, 0.01–0.02] [4], and the incidence of the composite outcome of hospitalization or death was 0.6% [95%CI, 0.4–0.7] [5]. In this study, the 30-day all-cause mortality for patients who received NMV-r was similar to those in the previous studies [4, 5]. Although the proportion of patients requiring oxygen therapy or ventilatory support was not reported in the previous studies [4, 5], the proportion of outcomes including hospitalization (i.e. less severe outcomes) in these studies [4, 5] was lower than the proportion of patients requiring oxygen therapy in this study. This may be due to the differences in patient backgrounds and the inclusion of inpatients in this study. Although MOV demonstrated no effectiveness in general patient populations with adequate vaccination status [6, 7], some observational studies including solid organ transplant recipients, patients with hematological malignancies, and patients aged ≥ 65 years with adequate vaccination status have demonstrated the effectiveness of MOV in reducing the rate of hospitalization or death [35–37]. In these studies, the mortality for patients who received MOV were 5.2% [95%CI, 1.9–10.9] and 1.2% [95%CI, 0.9–1.6], respectively [36, 37], indicating similar results in patients receiving MOV in this study. The proportion of patients requiring oxygen therapy or ventilatory support was not reported in these studies [36, 37]. In the present study, patients who received MOV had more risk factors, such as aged ≥ 65 years or prior solid-organ transplant, than patients who received NMV-r, as a result of the selection of the medication for COVID-19 according to the contents of DDIs. The mortality in the present study was similar to those in the previous studies [36, 37] that showed effectiveness in reducing mortality, suggesting that MOV may be a viable treatment option to improve clinical outcomes in patients with high-risk factors for whom NMV-r is unavailable due to DDIs. Although the mortality in patients who received each drug was similar to previous studies [4, 5, 36, 37], it is unclear whether the management system improved clinical outcomes in the overall patient population, because we could not compare the situation without the present management system. Further studies are warranted to determine the impact of the system that promotes the use of NMV-r in appropriate patients while avoiding problematic DDIs on clinical outcomes in patients with COVID-19.

This study has several limitations. First, this is a single-center, retrospective, observational study, and the distribution of DDIs may not represent various medical facilities across Japan. Our facility is a university hospital, with a higher proportion of patients with risk factors for severe COVID-19 than the general population. We could not evaluate the effectiveness of our system in DDI management with comparators. Additionally, some patients could not be evaluated for concomitant medications prescribed at other hospitals and clinical outcomes after treatment. Although the dosage of NMV-r should be assessed using eGFR (mL/min), which is not normalized by body surface area, we assessed it using eGFR (mL/min/1.73m^2^) because the height or weight of some patients was retrospectively unavailable. Second, the recommended DDI management in our constructed list was based on the NIH DDI guidelines [8] and the JSPHCS DDI guidance [24], nevertheless, the recommendations for several medications differed in their classification. This is because we considered that frequent visits and laboratory tests in outpatients with mild COVID-19 were difficult due to isolation measures, as previously mentioned. Third, this study did not evaluate adverse events due to DDIs. Future studies are needed to evaluate whether appropriate DDI management could reduce adverse events due to DDIs with NMV-r.

In conclusion, our study suggested the effectiveness of the management system for the appropriate use of NMV-r in the DDI management. Our management system promoted the use of appropriate antivirals based on the classification of DDIs with NMV-r and the implementation of recommended DDI management. Although 60% of the eligible patients had problematic DDIs with NMV-r, 22% of the patients required consideration for a change of NMV-r due to DDIs. Methods for the appropriate DDI management should be established and expanded to ensure the appropriate use of NMV-r and improve COVID-19 treatment outcomes.

### Supplementary Information

Supplementary Material 1.

## Data Availability

The datasets used and/or analyzed during the current study are available from the corresponding author on reasonable request.

## Abbreviations

BIC/TAF/FTC: Bictegravir/tenofovir alafenamide/emtricitabine
CI: Confidence interval
COVID-19: The coronavirus disease 2019
CYPs: Cytochrome P450 enzymes
DDIs: Drug-drug interactions
eGFR: Estimated glomerular filtration rate
HIV: Human immunodeficiency virus
HMG-CoA: 3-Hydroxy-3-methylglutaryl-coenzyme A
JSPHCS: Japanese Society of Pharmaceutical Health Care and Sciences
MOV: Molnupiravir
NIH: National Institutes of Health
NMV-r: Nirmatrelvir/ritonavir
P-gp: P-glycoprotein
PIMs: Potentially inappropriate medications
SARS-CoV-2: Severe acute respiratory syndrome coronavirus 2
TDM: Therapeutic drug monitoring
