# Supplementary material for: Evaluation of the drug-drug interactions management system for appropriate use of nirmatrelvir/ritonavir: a retrospective observational study
Source: J Pharm Health Care Sci. 2024 Sep 3;10:54. doi: 10.1186/s40780-024-00376-4 (PMC11370042; doi:10.1186/s40780-024-00376-4)
Supplement: Supplementary file 1 — Supplementary Material 1. [file 40780_2024_376_MOESM1_ESM.docx]

Table S1 Baseline characteristics of the patients who received each medication

| Category^a^ | NMV-r group (n = 126) | MOV group (n = 115) |
| --- | --- | --- |
| Age (years) | 55.5 [42.0–70.0] | 65 [51.5–75.5] |
| Female | 61 (48.4%) | 51 (44.3%) |
| eGFR (mL/min/1.73 m^2^) | 73.9 [63.8–87.3] | 60.5 [42.8–82.5] |
| Unknown | 5 | 4 |
| Number of severity risk factors | 2 [1.0–3.8] | 4 [2.0–5.0] |
| ≥ 65 years of age | 46 (36.5%) | 58 (50.4%) |
| Malignancy | 62 (49.2%) | 36 (31.3%) |
| Chronic respiratory disease | 13 (10.3%) | 15 (13%) |
| Chronic kidney disease | 25 (19.8%) | 55 (47.8%) |
| Diabetes mellitus | 19 (15.1%) | 34 (29.6%) |
| Hypertension | 40 (31.7%) | 64 (55.7%) |
| Hyperlipidemia | 23 (18.3%) | 48 (41.7%) |
| Cardiovascular disease | 22 (17.5%) | 31 (27.0%) |
| Cerebrovascular disease | 3 (2.4%) | 12 (10.4%) |
| Body Mass Index ≥ 30 kg/m^2^ | 9 (7.1%) | 6 (5.2%) |
| Smoking (smoking within the past 30 days and more than 100 cigarettes in a lifetime) | 15 (11.9%) | 5 (4.3%) |
| Prior solid-organ transplant | 0 (0.0%) | 17 (14.8%) |
| Late pregnancy | 3 (2.4%) | 0 (0.0%) |
| Use of immunosuppressants (including cancer chemotherapy) | 52 (41.3%) | 50 (43.5%) |
| HIV infection (CD4 < 200 cells/mm^3^) | 1 (0.8%) | 0 (0.0%) |
| Hospitalized patients | 27 (21.4%) | 17 (14.8%) |
| Vaccination status |  |  |
| 0–1 | 17 (13.5%) | 27 (23.5%) |
| ≥ 2 | 91 (72.2%) | 79 (68.7%) |
| Unknown^d^ | 18 (14.3%) | 9 (7.8%) |

eGFR, estimated glomerular filtration rate; HIV, human immunodeficiency virus; MOV, molnupiravir; NMV-r, nirmatrelvir/ritonavir

^a^Median [min–max] or n (%)
